# Linking Cell Architecture to Mitochondrial Signaling in Neurodegeneration: The Role of Intermediate Filaments

**DOI:** 10.3390/ijms262411852

**Published:** 2025-12-08

**Authors:** Emanuele Marzetti, Rosa Di Lorenzo, Riccardo Calvani, Hélio José Coelho-Júnior, Francesco Landi, Vito Pesce, Anna Picca

**Affiliations:** 1 Fondazione Policlinico Universitario “Agostino Gemelli” IRCCS, L.go A. Gemelli 8, 00168 Rome, Italyriccardo.calvani@unicatt.it (R.C.);; 2 Department of Geriatrics, Orthopedics and Rheumatology, Università Cattolica del Sacro Cuore, L.go F. Vito 1, 00168 Rome, Italy; 3 Department of Biosciences, Biotechnologies and Environment, Università degli Studi di Bari Aldo Moro, Via Edoardo Orabona 4, 70125 Bari, Italy; rosa.dilorenzo@uniba.it (R.D.L.); vito.pesce@uniba.it (V.P.); 4 Department of Medicine and Surgery, LUM University, Str. Statale 100, 70010 Casamassima, Italy

**Keywords:** axonal transport, cell architecture, cell quality, cytoskeleton, mitochondrial dynamics, mitochondrial quality, mitophagy, neuron, neurofilaments, reactive oxygen species

## Abstract

Mitochondrial dysfunction is a pivotal contributor to neurodegeneration. Neurons heavily rely on mitochondrial oxidative metabolism and therefore need highly efficient quality control mechanisms, including proteostasis, mitochondrial biogenesis, fusion–fission dynamics, and mitophagy, to sustain bioenergetics and synaptic function. With aging, deterioration of mitochondrial quality control pathways leads to impaired oxidative phosphorylation, excessive reactive oxygen species generation, calcium imbalance, and defective clearance of damaged organelles, ultimately compromising neuronal viability. Pathological protein aggregates, such as α-synuclein in Parkinson’s disease, β-amyloid and tau in Alzheimer’s disease, and misfolded superoxide dismutase 1 and transactive response DNA-binding protein 43 in amyotrophic lateral sclerosis, further aggravate mitochondrial stress, establishing self-perpetuating cycles of neurotoxicity. Such mitochondrial defects underscore mitochondria as a convergent pathogenic hub and a promising therapeutic target for neuroprotection. Intermediate filaments (IFs), traditionally viewed as passive structural elements, have recently gained attention for their roles in cytoplasmic organization, mitochondrial positioning, and energy regulation. Emerging evidence indicates that IF–mitochondria interactions critically influence organelle morphology and function in neurons. This review highlights the multifaceted involvement of mitochondrial dysfunction and IF dynamics in neurodegeneration, emphasizing their potential as targets for novel therapeutic strategies.

## 1. Introduction

Mitochondrial dysfunction plays a central role in neurodegeneration and the onset of age-associated neurological disorders [1]. Neurons show exceptionally high energy demands and rely heavily on intact mitochondrial quality control (MQC) systems, including proteostasis, mitochondrial biogenesis, fusion–fission dynamics, and mitophagy, to support cellular homeostasis and synaptic activity [2]. During aging, MQC pathways deteriorate, resulting in impaired oxidative phosphorylation, increased reactive oxygen species (ROS) generation, calcium dyshomeostasis, and defective clearance of damaged mitochondria. These alterations disrupt synaptic signaling, axonal transport, and neuronal viability.

Pathological protein aggregates, such as α-synuclein in Parkinson’s disease (PD), β-amyloid and tau in Alzheimer’s disease (AD), and misfolded superoxide dismutase 1 (SOD1) and transactive response DNA-binding protein 43 (TDP-43) in amyotrophic lateral sclerosis (ALS), have been found to further exacerbate mitochondrial stress and bioenergetic failure, creating a self-reinforcing cycle of neuronal injury [3]. These mitochondrial defects highlight a convergent pathogenic mechanism underlying diverse neurodegenerative diseases, underscoring mitochondria as a promising therapeutic target for disease modification and neuroprotection [1,3,4,5,6].

Intermediate filaments (IFs) have been long considered secondary components of the cytoskeleton, especially when compared to microtubules and actin filaments, and often overlooked as regulators of cell energetics and mitochondrial activity. However, the growing interest in IF biology and the results collected in recent years have attributed to IFs a crucial role in cytoplasmic organization and cell adhesion beyond their structural role and protective function against mechanical stress. Several lines of evidence indicate that IFs are involved in mitochondrial anchoring, positioning, and function and that IF–mitochondria interactions in neurons are pivotal for regulating organelle morphology and organization [7]. However, a comprehensive synthesis of how IF remodeling directly interfaces with mitochondrial signaling, organelle dynamics, and quality-control pathways across major neurodegenerative diseases is lacking. Herein, we sought to: (1) integrate molecular, biophysical, and organelle-level perspectives to highlight IFs as active regulators, rather than merely markers, of mitochondrial health; (2) compare specific mechanisms across neurodegenerative diseases with an emphasis on shared cytoskeletal–mitochondrial coupling; and (3) outline forward-looking questions and mechanistic gaps to identify emerging research directions. By combining established findings with new conceptual connections and future perspectives, this review provides an updated framework for understanding how IF dysregulation contributes to mitochondrial vulnerability in neurodegeneration.

## 2. Intermediate Filaments: Ultrastructure and Functional Roles in Neurons

IFs are a large family of nuclear and cytoplasmic proteins with rope-like features and without polarity. Cytoplasmic IFs can be classified into three major categories: types I and II keratin filaments found in epithelial cells, type III vimentin and vimentin-like filaments in connective tissue, muscle, and neuroglial cells, and type IV neurofilaments (Nfls) characteristic of neurons.

**Figure 1:**
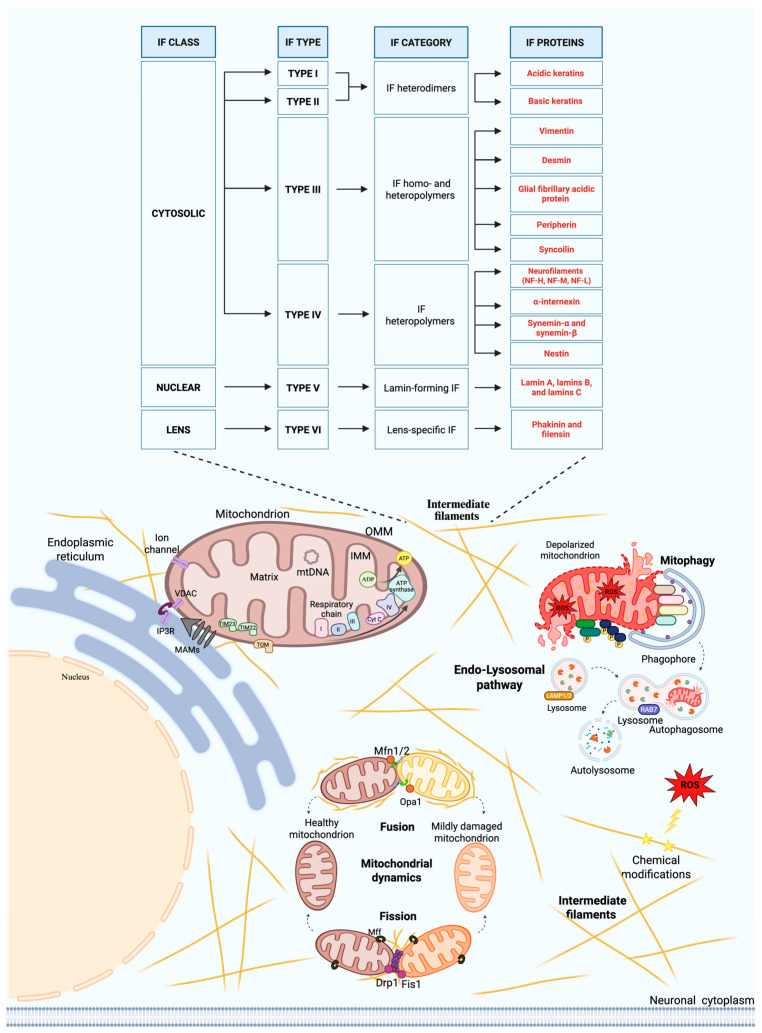
Main classes of intermediate filaments and their interactions with cellular components relevant to neurodegeneration. Abbreviations: Cyt C, cytochrome C; Drp1, dynamin-related protein 1; Fis1, fission protein 1; IF, intermediate filament; IMM, inner mitochondrial membrane; IP3R, inositol triphosphate receptor; LAMP, lysosome-associated membrane protein; MAMs, mitochondria-associated membranes; Mff, mitochondrial fission factor; Mfn, mitofusin; mtDNA, mitochondrial DNA; OMM, outer mitochondrial membrane; Opa1, optic atrophy 1; RAB7, Ras-related protein in brain 7; ROS, reactive oxygen species; TIM, translocase of the inner mitochondrial membrane; TOM, translocase of the outer mitochondrial membrane; VDAC, voltage-dependent anion channel. Created in Created in BioRender. Picca, A. (2025) https://BioRender.com/98g1ghl.

Keratin filaments (types I and II) comprise acidic and basic keratins organized into heterodimers. These keratin pairs are organized in different epithelial structures: as simple keratins in single-layer epithelia, as epidermal keratins in stratified epithelia, and as structural keratins in hair and nails [8]. Type III IFs include vimentin and several vimentin-related members (i.e., desmin, glial fibrillary acidic protein (GFAP), peripherin, and syncoilin), which can form both homo- and heteropolymers [9]. Among these, GFAP is expressed by glial cells, while syncoilin is found in muscle tissue [10]. Peripherin is produced by neurons of the peripheral nervous system (PNS) and contributes to the assembly of NFl together with type IV IFs. Type IV proteins include high molecular weight Nfl (NF-H, 200 kDa), medium molecular weight Nfl (NF-M, 160 kDa), low molecular weight Nfl (NF-L, 68 kDa), and α-internexin in neurons [11]. Additionally, synemin-α and synemin-β are expressed in astrocytes, neurons, and muscle cells, whereas nestin is found in stem and endothelial cells [11,12,13]. Keratins and vimentin constitute structural components of the cell cortex [14,15], where they contribute to form a transcellular network that connects the plasma membrane to the nuclear compartment, thereby supporting the architecture of individual cells and tissues [16,17,18]. IFs are also integral components of the nucleus, represented by type V intranuclear filaments. This class consists of six lamin proteins encoded through alternative splicing of the LMNA, LMNB1, and LMNB2 genes [19]. Nuclear lamins form a three-dimensional network lining under the inner nuclear membrane, thereby supporting and shaping the nuclear envelope while providing anchoring sites for chromatin, which is crucial for preserving nuclear shape/structure and genome stability [20]. Finally, type VI IFs, encompassing phakinin and filensin, are specifically expressed in the eye lens and display structural properties distinct from canonical IF proteins [21] (Figure 1).

**Figure 2:**
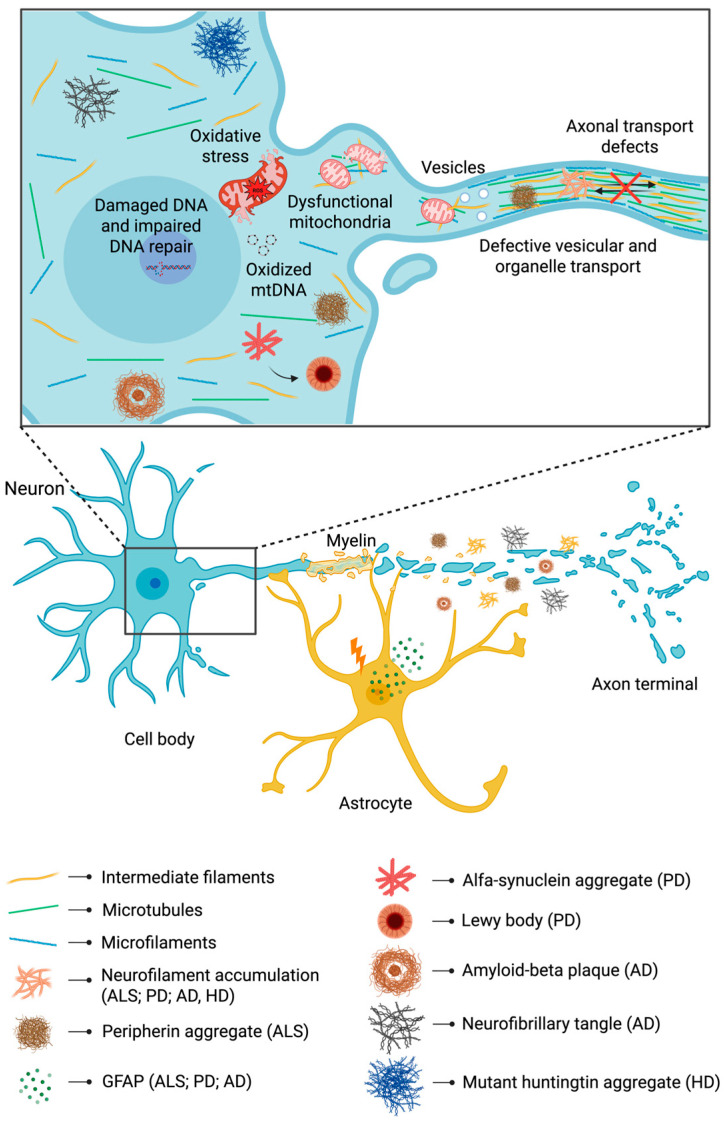
Schematic representation of pathological intermediate filaments aggregates and mitochondrial derangements in Alzheimer’s disease (AD), amyotrophic lateral sclerosis (ALS), Huntington’s disease (HD), and Parkinson’s disease (PD) neurodegeneration. Abbreviation: GFAP, glial fibrillary acidic protein. Created in BioRender. Picca, A. (2025) https://BioRender.com/vumdzc7.

Beyond their well-established role as structural and mechanosensory elements, IFs contribute to vital cellular processes, including growth, differentiation, regulation of apoptosis, and migration [22]. Yet, the molecular mechanisms underlying these different functions remain only partially understood. Currently, hypotheses regarding their mode of action include: (1) direct molecular interactions with specific cellular components; (2) bidirectional signaling between IFs and other cellular compartments; and (3) functional sequestration of cellular components by the IF networks [23]. Among the most studied cellular interactions with IFs in neurons is that of mitochondria that likely influences organelle morphology, subcellular localization, and function both directly and indirectly [23,24]. Since mitochondria accumulate in regions with high energy demand, their proper distribution depends on complex transport and anchoring systems (Figure 2).

While the roles of microtubules and actin in these processes are well established [25], the role of IFs is less clear, partly because their apolar structure does not support directional transport [26]. Nonetheless, increasing evidence indicates that IFs are involved in mitochondrial anchoring, positioning, and function [7], opening new perspectives in understanding neurodegenerative, muscular, and metabolic disorders [27,28].

## 3. Intermediate Filaments and Mitochondria Interactions

### 3.1. Vimentin and Desmin

Vimentin, desmin, and Nfls are key IF proteins interacting with mitochondria [27,29,30,31]. Mitochondrial morphology is shaped by mechanical interactions between microtubules (which promote elongation), actin, and vimentin (which modulate curvature) [32].

Vimentin belongs to the type III IF protein family and is predominantly expressed in many mesenchymal-derived cells, such as endothelial cells, fibroblasts, macrophages, osteoblasts, and vascular smooth muscle cells, as well as in most neural progenitor cells [24]. Recent findings also highlighted the role of vimentin in peripheral nerve development and regeneration [33,34], and sensory organ regeneration [35]. Vimentin downregulation in CV-1 in origin, carrying SV40 (COS7) cells has shown to induce mitochondrial fragmentation [7]. Vimentin–mitochondria interactions have been described both as direct [7] and indirect via IF-associated proteins like plectin, an essential cytolinker protein [36,37]. Such interactions influence mitochondrial mobility and cytoplasmic anchoring [7,38,39,40]. In particular, the plectin isoform 1b has been identified as a mediator of the vimentin–mitochondria link [37,41]. In plectin 1b knockout mice, mitochondria appear more elongated while their motility is not impaired [36], likely indicating that not all mitochondrial functions mediated by IFs depend on this protein. In the absence of intermediary proteins, vimentin interacts directly with mitochondria exhibiting active respiration, possibly to enhance localized ATP production [42]. Such mitochondria appear as stationary and have a higher membrane potential than mobile organelles [42]. Indeed, vimentin seems to be involved in the regulation of membrane potential [42,43]. Vimentin-null mouse embryonic fibroblasts have mitochondria with higher motility and lower potential, which both are reversible upon reintroduction of wild-type vimentin [42]. It has been hypothesized that vimentin’s N-terminal domain interacts with a yet unidentified receptor on the outer mitochondrial membrane (OMM) [42]. Phosphorylation of vimentin via Rac family small GTPase 1 (Rac1) at the mitochondrial binding site in polarized fibroblasts induces spatial differentiation of mitochondria with relocation of those with lower potential membrane and a reduced ATP production at the cell front compared with those of the rear part [39,44]. Moreover, vimentin deletion in catecholaminergic Cath.a-differentiated (CAD) neurons has been found to reduce mitochondrial membrane potential, which could be restored by reintroducing wild-type vimentin at the mitochondrial binding site [43].

Desmin, another main component of IF proteins, is primarily expressed in striated and smooth muscles, where it preserves mitochondrial and nuclear integrity. The L345P desmin mutation induces cytoskeletal aggregates and causes severe mitochondrial abnormalities and calcium dysregulation in skeletal and cardiac muscles [45]. Desmin-deficient mitochondria are more susceptible to calcium overload [46], exhibit matrix vacuolization, rarefied cristae, and giant morphology [30,45]. In the soleus muscle, desmin loss reduces mitochondrial ADP uptake and respiratory rate, due to impaired coupling between creatine kinase and the adenine nucleotide translocator (ANT) [30]. ANT and the voltage-dependent anion channel (VDAC) form a functional complex for ATP/ADP exchange between the matrix and intermembrane space [47]. Single knockout of either vimentin or desmin in BHK21 cells did not affect mitochondrial potential, while double knockout significantly did, likely indicating a cooperative role in mitochondrial regulation [47]. However, our understanding of their role in the regulation of mitochondrial bioenergetics and physiology remains incomplete.

### 3.2. Glial Fibrillary Acidic Protein

Glial fibrillary acidic protein (GFAP) is the main IF protein in astrocytes, where it plays a pivotal role in maintaining cytoskeletal integrity, cell shape, and intracellular organization [48]. GFAPα can self-assemble in cell-free systems [49], but to form intermediate filaments (IFs) in astrocytes, it requires the presence of vimentin [50]. GFAP can also associate with the IF proteins nestin and synemin and form heterodimers [51]. Interaction with vimentin allows GFAP to form a dynamic filamentous network that supports mechanical stability, intracellular transport, and organelle positioning [48]. This network is essential not only for structural support but also for modulating signaling pathways and cellular metabolism. GFAP closely associates with organelles, including lysosomes and mitochondria, acting as a scaffold that influences organelle localization and interaction [52]. GFAP filaments interact with membrane-associated proteins such as lysosome-associated membrane protein 2A (LAMP-2A), thereby linking the cytoskeleton to autophagic and lysosomal compartments, which in turn regulate mitochondrial turnover and quality control [52,53]. GFAP–vimentin networks also govern trafficking interfaces with neurons: astrocytic endocytosis of the Notch ligand Jagged1 and downstream Notch signaling require these filaments, linking the IF scaffold to membrane trafficking and metabolic demands that engage mitochondria [54]. At the mitochondrial level, astrocytes use filament-supported transport systems to position mitochondria in fine processes, matching local ATP/Ca^2+^ buffering to synaptic activity. Experimental manipulation of mitochondrial motility shows reciprocal coupling between mitochondrial dynamics and astrocyte Ca^2+^ signals [55], and Miro1-dependent mechanisms place mitochondria near active synapses within astrocytic processes [56]. Under pathological conditions, such as reactive astrogliosis or Alexander disease (AxD), GFAP overexpression or mutation leads to formation of Rosental fibers (aggregates of ubiquitinated GFAPs, αB-crystallin, and heat shock protein 27) in astrocytes, filament disorganization, mitochondrial mislocalization, and impaired organelle crosstalk [48,57]. This can result in altered mitochondrial dynamics, disrupted energy metabolism, and increased oxidative stress, which contribute to astrocytic dysfunction and exacerbate neuronal vulnerability. Several studies have reported that, in mammals, mitochondria can undergo intercellular transfer [58], not only promoting the survival and recovery of damaged cells but also contributing to tumor cell metastasis and chemoresistance [59,60]. The transfer of mitochondria between astrocytes, and between astrocytes and neurons, is a dynamic process mediated by CD38/cyclic ADP-ribose signaling and mitochondrial Rho GTPases (MIRO1 and MIRO2). Mutations associated with AxD (R79C, R239C) in the GFAP gene of astrocytes differentiated from mutant human pluripotent stem cells have been shown to impair mitochondrial transfer from astrocytes and reduce CD38 expression, indicating a potential pathogenic mechanism in AxD [58]. In glioblastoma cell communication, GFAP acts as a structural component of tunneling nanotubes (TNTs), long plasma membrane and F-actin bridges connecting distant cells, which allow intercellular transfer of cellular components [61]. Under oxidative stress and pro-apoptotic stimuli, GFAP expression increases in TNTs and co-localizes and interacts with functional mitochondria during intercellular transfer, both at the closed ends of GFAP-positive TNTs and within recipient cells [61]. Overall, GFAP acts as a structural and functional integrator between the cytoskeleton, mitochondria, and intercellular communication in astrocytes. Under physiological conditions, it supports neuronal homeostasis through spatial control of organelles, calcium handling, and metabolic support. In contrast, GFAP mutations or overexpression lead to filament disorganization, mitochondrial mislocalization, and increased oxidative stress. Disruption of this interplay represents an important mechanism linking astrocytic pathology to neuronal dysfunction.

### 3.3. Neurofilaments

Nfls are the most prominent IFs in vertebrate neurons. These proteins are highly concentrated along axons, where they contribute to their structural stability [62]. Nfl expression varies throughout neuronal development: NF-L associates with α-internexin (class IV IF) or peripherin (type III IF) during differentiation; NF-M supports axonal elongation; and NF-H contributes to radial growth and myelination [63,64]. NFls form a stable yet elastic network that influences microtubule dynamics, mitochondrial distribution, and synaptic transmission [27]. At synaptic terminals, mitochondria not only produce ATP but also buffer Ca^2+^, thus regulating signal transmission. Mitochondrial transport along axons is mediated by microtubules and actin filaments. However, NF-L overexpression in cultured neurons has been shown to impair mitochondrial transport. In mice, NF-H mutants lacking the terminal domain do not show altered mitochondrial distribution, while full-length NF-H overexpression induces perinuclear mitochondrial aggregation [65,66]. The phosphorylated C-terminal domain of NF-H forms extended side arms that organize IFs within axons [67,68]. In both NF-H and NF-M, this domain appears to compete with microtubule-associated protein 2 (MAP2) for binding to VDAC, potentially affecting mitochondrial membrane potential [69]. Notably, stationary mitochondria with high membrane potential tend to bind phosphorylated NF-L [31]. In NF-L knockout mice, mitochondrial motility increases and fusion decreases [70,71]. Peripherin overexpression in these mice leads to abnormal retrograde axonal mitochondrial transport and cytoskeletal aggregates [71]. NF-L mutations can disrupt the neurofilament network, leading to axonal degeneration and mitochondrial accumulation in cell bodies [72]. These mutations also interfere with mitofusin 2 (Mfn2) protein, which regulates mitochondrial fusion and transport via the Miro/Milton complex [23,73].

### 3.4. Peripherin

Peripherin is a less characterized type III IF protein. It has a molecular weight of 58 kDa and shows strong sequence homology with other type III IFs but not with Nfl proteins [74,75]. The peripherin gene consists of nine exons interspersed with eight introns and shows ~90% nucleotide sequence conservation between humans and mice [28]. The divergence between species resides in 18 amino acid residues [76]. Multiple isoforms exist due to alternative splicing: in mice, Per 58 (dominant), Per 45, Per 56, and Per 61 [77,78]; in humans, Per 61 is absent, but Per 28 and Per 32 are expressed [28,79]. The term peripherin refers to its expression in neurons of the PNS, including motor neurons [80,81,82]. Peripherin also interacts with Nfl subunits [83], and its axonal abundance depends on NF-L expression [84]. In NF-L-deficient mice, peripherin overexpression leads to pathological IF inclusions and early motor neuropathy, a phenomenon linked to ALS pathology [85]. Overexpression of NF-H can counteract this phenomenon by sequestering peripherin and reducing toxic aggregates [86]. Like other IFs, peripherin also interacts with microtubules via kinesin and dynein, supporting axonal transport [87,88].

Peripherin plays a key role in neuronal growth, especially during axonal development and after injury [28,48,89] and it has recently been recognized as a biomarker of axonal damage in PNS diseases [90,91]. It is able of self-assembling into filamentous networks or co-assembling with other intermediate filaments, including syncoilin and NF-L [92]. In rat pheochromocytoma (PC12) cells, peripherin deficiency impairs neurite initiation, extension, and maintenance [93], underscoring its vital role in neuronal architecture. However, the lack of peripherin does not hinder the development of long, myelinated neurons as shown in knockout mice exhibiting normal development. Nonetheless, unmyelinated sensory axons in the L5 dorsal roots are found to be quantitatively reduced. This deficit is compensated by an upregulation of α-internexin in motor neurons, suggesting that peripherin contributes to the growth of a specific subset of sensory neurons [94], including those implicated in contacts with outer hair cells of the cochlea [95,96].

Peripherin is involved in several processes related to neurodegeneration. Soluble β-amyloid precursor protein (sAPP), a marker of AD, after cleavage, associates with perinuclear peripherin and is transported toward axon terminals along with proteins like TDP-43, fused in sarcoma (FUS), and SOD1 [97,98]. Removal of peripherin reduces TDP-43 accumulation in neurite terminals [98]. IF disorganization, including peripherin, can affect mitochondrial distribution and function [99]. In ALS neurons, peripherin aggregates often accompany dysfunctional mitochondria, though no direct interaction has been proven. Aggregation correlates with apoptosis [100], especially in cells with high protein kinase C epsilon (PKCε) levels. PKCε promotes peripherin aggregation and cell death through its C1b domain, while downregulating PKCε or co-expressing the domain prevents these effects [101]. Other kinases also modulate peripherin: protein kinase B (AKT) phosphorylates peripherin’s head domain, promoting nerve regeneration [102], and PKCα supports filament assembly in an ATP-dependent manner [103]. Various growth factors, including interleukin-6, leukemia inhibitory factor, fibroblast growth factor, and especially nerve growth factor (NGF), influence protein phosphorylation and its dynamic state [104,105,106].

## 4. Physiological Roles of Intermediate Filaments and Mitochondrial Crosstalk in Neurons

Mitochondria serve multiple roles within the cell also through establishing multiple contacts with cell components some of which can be mediated by IFs. While several lines of evidence indicate that IF–mitochondria physical associations are involved in regulating organelle positioning and/or trafficking [42,43], they also contribute to maintaining membrane potential and bioenergetic integrity.

### 4.1. Intermediate Filaments in Mitochondria–Endoplasmic Reticulum Interactions

Mitochondria interact with the endoplasmic (or sarcoplasmic) reticulum (ER) through mitochondria-associated membranes (MAMs). Alterations in ER–mitochondria distance affect mitochondrial spatial organization [107]. MAMs are critical for lipid trafficking and Ca^2+^ transfer from the ER to mitochondria via the inositol trisphosphate (IP3) receptor–VDAC complex [108,109,110]. Mild mitochondrial Ca^2+^ elevation promotes ATP production, while excessive Ca^2+^ levels can trigger autophagy and apoptosis [111]. B-cell lymphoma 2 (BCL-2) family proteins, by interacting with VDAC, regulate cytochrome C release and protect mitochondria from calcium overload [112]. MAMs are also crucial for importing lipid precursors. For instance, cardiolipin biosynthesis within the mitochondria requires phosphatidic acid delivered from the ER. Such lipid exchange occurs at MAMs. Thus, ER–mitochondria contacts support mitochondrial phospholipid remodeling and membrane curvature [107,113]. The keratin-filament binding protein trichoplein/mitostatin has been shown to modulate ER–mitochondria contacts [114,115], potentially explaining lipid imbalances in keratin-deficient cells [115].

### 4.2. Intermediate Filaments and Mitochondrial Dynamics

Mitochondrial dynamics, achieved via fusion and fission events, are essential for maintaining mitochondrial morphology and function [27]. Mitochondrial fission allows separation of damaged portions of the organellar network, thereby preserving the healthy portions. Mitochondrial fusion, on the other hand, allows functional recovery via merging with new mitochondria generated through biogenesis. Fission is driven by dynamin-related protein 1 (DRP1), recruited to the OMM by mitochondrial fission 1 protein (FIS1) e mitochondrial fission factor (MFF1) [116,117,118]. Fusion involves MFN 1 and 2 on the OMM and optic atrophy protein 1 (OPA1) on the inner membrane [119,120]. IFs actively contribute to this balance. Mutations in the Nefl gene, encoding NF-L, impair mitochondrial dynamics and are linked to Charcot–Marie–Tooth (CMT) disease. In Nefl knockout motor neurons, mitochondria are shorter, fusion is slower, and motility is increased. These defects can be corrected only by wild-type NF-L and not MFN2 overexpression, confirming the crucial role of Nfls. Mutations in NF-L causing CMT disease, such as NFLQ333P and NFLP8R, mimic these effects and further disrupt mitochondrial motility in later stages [70]. NFLQ333P directly interferes with MFN2, reducing fusion rates. In contrast, in retinal pigment epithelial cells, keratin 8 interacts with mitochondria under oxidative stress, promoting mitophagy via fission to protect mitochondrial integrity and cell survival [121]. Ultimately, the fusion–fission balance not only shapes mitochondria but also determines the architecture of the mitochondrial network, influencing interactions with other organelles and the cytoskeleton [122,123].

### 4.3. Intermediate Filaments and Mitochondrial Redox Regulation

IFs are highly responsive to oxidative stress and participate actively in redox signaling, triggering cytoskeletal rearrangements in response to reactive oxygen (ROS), nitrogen (RNS), and lipid peroxidation [124,125,126]. Modifications of nucleophilic residues on IFs, such as vimentin’s cysteines, result in functional changes that accumulate with age and/or in neurodegenerative conditions [127,128,129]. Given its close mitochondrial interactions and role in biomechanical signaling, vimentin is also involved in the regulation of cellular stress. Mitochondria are the main intracellular source of ROS, and their functional impairment triggers oxidative stress. IFs support mitochondrial homeostasis; in immune cells, vimentin has been shown to limit pro-inflammatory cytokine and ROS production, and to affect mitochondrial gene expression [130]. Loss of vimentin or desmin leads to mitochondrial dysfunction and increased oxidative stress in various models [131,132], worsening the progression of genetic diseases such as those caused by desmin and glial fibrillary acidic protein (GFAP) mutations [133,134], which amplify oxidative damage. In giant axonal neuropathy, a genetic disorder caused by defective proteolysis, neurofilament accumulation blocks mitochondrial transport and increases oxidative stress [135].

### 4.4. Intermediate Filaments and the Endo-Lysosomal System

Intermediate filaments have also been involved in regulating late endocytic trafficking. Because endo-lysosomal pathways provide a degradative route for effective mitophagy, disruptions in IF-dependent lysosomal positioning or trafficking can directly compromise MQC, thereby linking endo-lysosomal dysfunction to broader IF–mitochondria axis. However, no evidence currently exists on whether IFs regulate mitochondrial multivesicular body formation and/or mitochondrial turnover via mitochondria-derived vesicles.

The adaptor protein 3 (AP-3) complex, which is known to participate in the sorting of proteins within the endo-lysosomal system, interacts with vimentin [136]. Furthermore, studies have shown that altered vimentin expression disrupts the subcellular localization of both AP-3 and lysosomes, as well as the expression of LAMP-1 and LAMP-2, both essential for autophagy regulation [137]. AP-3 has also been found to interact with peripherin and, given the structural similarity between vimentin and peripherin, it has been hypothesized that peripherin may contribute to regulating late endocytic trafficking [136]. This hypothesis is in line with recent work in mouse Neuro2a neuroblastoma cells, in which peripherin silencing affects the quantity, positioning, and activity of lysosomes that move from the perinuclear region to the cell periphery [138]. In this neuronal cell model, peripherin silencing has been shown to inhibit the epidermal growth factor receptor (EGFR) degradation and to affect also lysosomal biogenesis and autophagy flux [138]. These events were accompanied by decrease in protein expression of the transcription factors EB (TFEB) and E3 (TFE3) which are essential for regulating lysosomal function and autophagy [138]. This modulation may be mediated via interaction with RAB7A, a small GTPase that regulates trafficking to lysosomes and autophagosomes [139,140]. RAB7A expression indeed influences peripherin’s solubility and assembly [99].

Peripherin also affects fast axonal transport: its overexpression in dorsal root ganglion primary neuronal cultures increases anterograde lysosomal transport, while NF-L absence accelerates transport bidirectionally [71]. A yeast two-hybrid screen using mouse brain cDNA identified several peripherin interactors, including SIP30 (involved in exocytosis), Snapin, Cplx2 (vesicle trafficking), HGS (signal transduction), transcription regulators (HDAC2, zinc-finger proteins), and mitochondrial proteins like MFN1 [141,142]. These findings suggest broader roles for peripherin in processes like vesicular trafficking, transcriptional regulation, and mitochondrial metabolism, although further validation is required.

## 5. From Cytoskeletal Disturbance to Energy Deficits: Implications of Intermediate Filaments in Neurodegeneration

Neurodegenerative diseases, while presenting significant clinical heterogeneity, share a set of pathological features that suggest converging intracellular mechanisms culminating in neuronal vulnerability.

A recurring and compelling pattern is the disintegration or dysregulation of the cytoskeletal architecture, not merely at the level of microtubules and actin, but often involving IFs or proteins that interrelate with them. This structural destabilization, either in isolation or alongside pathological protein aggregation, commonly coexists with mitochondrial dysfunction, manifesting as bioenergetic deficits, oxidative stress, aberrant mitochondrial dynamics, impaired organelle trafficking, and failure of mitochondrial quality control. The concurrence of cytoskeletal derangement and mitochondrial dysfunction suggests that neuronal structural integrity is tightly interwoven with mitochondrial homeostasis; when the scaffolding fails, mitochondria may be “trapped”, mislocalized, functionally compromised, or inadequately cleared. Some of the putative shared mechanisms bridging cytoskeletal/IF derangement and mitochondrial impairment include: (1) physical obstruction by aggregates: accumulation of cytoskeletal-associated proteins or aggregates (e.g., neurofilaments, IFs, or misfolded proteins binding the cytoskeleton) can physically impede mitochondrial movement along axons or neurites, blocking their trafficking; (2) impaired organelle–cytoskeleton anchoring: IFs (such as vimentin) contribute to mitochondrial positioning and maintenance of membrane potential through scaffolding. Disruption of IF integrity may impair mitochondrial anchorage, leading to misdistribution, loss of local energy support, or displacement from synapses or nodes of high metabolic need; (3) failure of mitochondrial quality control/mitophagy: when damaged mitochondria are not removed (due to defective mitophagy or lysosomal dysfunction), they accumulate ROS, incur mtDNA damage, and develop structural defects (cristae loss, membrane permeabilization), accentuating cellular stress; (4) cascade propagation of injury: cytoskeletal defects retard organelle transport, leaving damaged mitochondria distal or near aggregates, instigating localized stress that propagates into axonal degeneration, synaptic failure, and ultimately neuronal loss (Table 1). We will discuss these aspects in the next sections and explore the intersection of these events in the setting of different conditions, including ALS, and major neurodegenerative conditions such as PD, AD, and Huntington’s disease (HD). Altogether, we propose a conceptual framework in which cytoskeletal integrity is essential for mitochondrial health, and vice versa, suggesting that therapeutic strategies targeting the cytoskeleton–mitochondria axis may be effective across multiple neurodegenerative conditions.

### 5.1. Amyotrophic Lateral Sclerosis

ALS is a rare neurodegenerative disease marked by axonal degeneration of upper and lower motor neurons across multiple regions of the spinal cord, leading to weakness, atrophy, paralysis, and ultimately respiratory failure [143,144]. Although the molecular mechanisms remain unclear, several converging pathological processes have been proposed, including abnormal peripheral inclusions, lysosomal/autophagy and mitochondrial dysfunction, and distal-to-proximal axonal dieback [145,146]. A hallmark of defective axonal transport and cytoskeletal disarrangements is the swelling of the axon initial segment, where vesicles, lysosomes, mitochondria, and IFs, especially neurofilaments and peripherin, accumulate [147].

Peripherin abnormalities are consistently observed in both familial and sporadic ALS [148]. Normally expressed at low levels, peripherin protein expression is upregulated in spinal motor neurons, likely as a regenerative attempt [89,149,150]. Immunostaining analyses reveals its abundance in spheroids, Bunina bodies, and Lewy body–like inclusions [148,150,151,152]. Overexpression models confirm its pathogenic role: transgenic mice develop age-related motor neuron degeneration [85], and cultured cells undergo apoptosis upon peripherin accumulation [153]. In PC12 cells, protein aggregates trap mitochondria and ER, activate caspases 9 and 3, and trigger mitochondrial apoptotic pathways [154]. These findings support that peripherin inclusions impair trafficking, disrupt ER–mitochondria communication, and contribute to neuronal death.

On the other hand, peripherin silencing alters lysosomal localization and function, disrupts EGFR degradation, blocks autophagic flux, and reduces TFEB and TFE3 protein levels [138], linking peripherin to lysosomal regulation. Since mitophagy relies on intact autophagy-lysosome systems [155], peripherin aggregation may exacerbate mitochondrial dysfunction, which is an early hallmark of ALS pathogenesis [156,157]. These aggregates also sensitize neurons to TNF-α released by activated microglia, thus amplifying neurodegeneration [153,158]. Perturbations in IF stoichiometry further modulate the disease. NF-L mRNA is reduced in ALS, and double knockout NF-L/peripherin overexpression accelerates ALS pathology [85,159]. In contrast, overexpression of peripherin and NF-H without NF-L prevents axonal inclusions [86,160]. Dysregulation of miR-105 and miR-9, which stabilize peripherin and NF-L mRNAs, may shift IF composition [161]. Peripherin gene mutations, including PRPH deletion (228delC), IVS8(−36insA), and missense variants p.D141Y and p.R133P, impair filament network assembly and have been found in ALS patients [162,163,164].

In ALS models with mutant SOD1, peripherin forms intraneuronal inclusions [85], with the Per-61 splice variant promoting aggregation and toxicity [165]. Additional isoforms (e.g., Per-28) also exhibit strong aggregative potential, disrupting transport and metabolism [78,79,86,166]. While peripherin deletion does not alter ALS onset [167], compensatory expression of α-internexin and vimentin occurs [94,168]. Mutant SOD1 toxicity involves misfolding, aggregation, and multiple neurodegenerative pathways, including oxidative stress, cytoskeletal abnormalities, and neuroinflammation [169,170].

Peripherin alterations also affect axonal transport of TDP-43 [171]. Overexpression slows neurofilament transport and restricts essential cargo movement, including mitochondria [166]. This dysfunction aligns with the “axonal dieback” pattern of ALS, where degeneration begins distally and progresses proximally [172,173]. Peripherin aggregation and mitochondrial dysfunction thus converge on disrupted axonal transport, limiting energy supply and structural integrity, and supporting neurodegeneration.

An important conceptual nuance in peripherin biology concerns also the distinction between pathogenic versus compensatory changes. While aberrant IF aggregation and misassembly clearly contribute to cytoskeletal instability, impaired organelle trafficking, and mitochondrial dysfunction, the upregulation of peripherin in ALS also indicates that IF remodeling can also arise as an attempted protective or regenerative response. While increased IF expression may initially promote axonal stabilization, enhance organelle anchoring, and/or buffer mechanical stress, yet persistent or excessive induction can lead to maladaptive filament accumulation and further disrupt cellular homeostasis. Thus, IF alterations may likely serve a continuum between compensation and pathology, with the eventual outcome determined by the magnitude, duration, and cellular context of the response. A clear definition of these dual roles is essential for interpreting IF changes across neurodegenerative diseases and for identifying which IF-modulating strategies may be therapeutically beneficial versus detrimental.

Although peripherin shows promise as a biomarker, several limitations currently restrict its clinical utility. Its detection sensitivity remains suboptimal with standard immunoassays, and even with ultrasensitive platforms, methodological standardization is lacking [174]. Peripherin’s predominant expression in the peripheral nervous system narrows its diagnostic scope compared to established axonal markers such as Nfl. Moreover, its kinetics and clearance are poorly understood, hindering the definition of reliable thresholds and temporal dynamics [174]. Potential biological variability and confounding peripheral conditions may also affect interpretation. Finally, longitudinal and outcome-correlated data are scarce, limiting its immediate translational value [174].

Given the release of GFAP from activated or damaged glial cells into the extracellular space [175,176] a role for this intermediate filament as a potential biofluid biomarker has been proposed. However, findings are inconsistent [177,178] and no significant differences have been found for circulating levels of GFAP in ALS patients compared to healthy controls [179,180]. Nonetheless, a recent study reported significantly higher plasma levels of GFAP compared to controls, with a greater increase in females than in males [181]. Importantly, AD co-pathology has a decisive impact on plasma GFAP levels in ALS patients; thus, plasma GFAP represents an accurate biomarker for detecting AD co-pathology in ALS, which may influence the cognitive phenotype [181].

### 5.2. Parkinson’s Disease

PD is a neurodegenerative disease PD involving the loss of dopaminergic neurons in the substantia nigra pars compacta. Among the pathological traits of the disease is the aggregation of α-synuclein (α-syn) forming Lewy bodies that interact with the cytoskeleton and disrupt intracellular dynamics. Of note, Lewy bodies contain neurofilament proteins (co-aggregation with α-syn) [182]. Recent studies show that α-syn inclusions also disrupt mitochondrial function in dopaminergic and cholinergic neurons: they downregulate mitochondrial gene expression, reduce ATP production, increase oxidative stress, and trigger neuronal death [183]. Although direct dismantling of classical intermediate filaments has not always been demonstrated, secondary cytoskeletal alterations are well documented. PD brains show reduced NF-L and NF-M expression in dopaminergic neurons of the substantia nigra, at both mRNA and protein levels, together with oxidative stress markers. Neurofilaments normally stabilize axonal structure and serve as tracks and anchors for organelle transport, including mitochondria. Their disorganization impairs mitochondrial distribution, particularly to distal axons and synapses, creating local energy failure [184]. Mitochondrial impairment in PD, especially at complex I, leads to excessive ROS production. ROS oxidizes and nitrates NF proteins, increasing their instability and aggregation tendency [184,185]. This creates a vicious cycle in which mitochondrial damage worsens NF pathology, which in turn further impedes organelle transport [185]. Mutations in NEFM gene have been reported in early-onset PD [186] and circulating levels of NF-L protein has been found to support PD phenotyping. In particular, NF-L levels are lower in idiopathic PD compared to atypical parkinsonism (MSA/PSP), but higher NF-L predicts PD dementia and tracks severity/progression [187,188]. Therefore, neurofilament depletion/dysregulation within dopaminergic systems weakens axonal caliber/maintenance and NF-L levels in biofluids reflects neuroaxonal damage and helps stratify PD versus mimics [189]. GFAP has been proposed as a potential predictive biomarker of cognitive decline in patients with PD, particularly in the tremor-dominant (TD) subtype [190]. Indeed, baseline levels in the cerebrospinal fluid (CSF) and longitudinal increases in GFAP are associated with greater episodic memory decline, reduced α-synuclein, and increased NF-L. Moreover, higher baseline GFAP levels have been found to predict the development of mild cognitive impairment (MCI) within four years, suggesting that GFAP could serve as a useful indicator of cognitive progression in PD, especially in the TD subtype [190]. The clinical value of a plasma biomarker panel for stratifying patients and predicting PD progression over a three-year follow-up has also been recently evaluated [191]. Among the biomarkers analyzed, NF-L and GFAP showed a strong correlation with baseline motor severity, confirming previous findings [192], while p-tau181 showed only a weak association with cognitive function [191]. Over time, NF-L emerged as the best predictor of both motor and cognitive progression, with only marginal contributions from GFAP and p-tau181 [191]. These results confirm the utility of NF-L as a biomarker of neuronal damage and suggest that alterations in GFAP and tau may reflect possible AD-type co-pathology in PD, though with limited prognostic value. Interestingly, a study found that in enteric glial cells (EGCs) of PD patients, a glial reaction occurs, characterized by overexpression and hypo-phosphorylation of GFAP, particularly its GFAPκ isoform, compared to healthy subjects and patients with atypical parkinsonism. These alterations indicate that enteric glia become specifically reactive in PD, suggesting a possible involvement of the enteric nervous system in the onset and progression of the disease [193]. In models of PD bearing LRRK2 mutations, mitochondrial defects and impaired mitophagy clearance have also been observed [194]. Hence, in PD the combination of proteotoxic aggregation and mitochondrial dysfunction, alongside cytoskeletal disorganization, even when not directly involving IFs, constitutes a conceptual parallel with ALS.

### 5.3. Alzheimer’s Disease

AD is a neurodegenerative disorder and a major cause of dementia. It is characterized by the accumulation of misfolded proteins in the brain, resulting in amyloid plaques and neurofibrillary tangles. This pathological buildup progressively leads to neuronal loss and brain atrophy.

Tau protein is a microtubule-associated protein that can influence neurofilaments and other IF networks via cytoskeletal disorganization. Pathological tau forms lead to destabilization of the cytoskeletal network, loss of axonal integrity, and impaired intracellular transport. Amyloid-β oligomers (Aβ), neurotoxic peptide aggregates, rapidly induce defects in mitochondrial transport along dendrites and, with prolonged exposure, mitochondrial fragmentation via DRP1 [195]. This indicates that cytoskeletal disruptions (microtubules transport) often precede or accompany mitochondrial deficits such as reduced oxidative phosphorylation, oxidative stress, and failure of respiratory chain function. In a recent study, mitochondrial components, such as SOD2 and DNase I, were analyzed in different biofluids of patients with AD and proposed as possible biomarkers for AD-related mitochondrial oxidative stress [196]. Circulating plasma phosphorylated tau, particularly p-tau217, has emerged as a more accurate and scalable biomarker for AD [197]. It correlates strongly with amyloid PET, offers high predictive value in cognitively impaired individuals, and enables earlier, less invasive diagnosis and risk stratification [197]. However, several limitations remain. Its performance in cognitively unimpaired populations is lower, and false positives may occur in non-AD tauopathies or mixed pathologies. Moreover, standardized thresholds and longitudinal data are still needed to guide clinical decision-making [197]. Astroglial activation plays a key role in AD. Cerebral GFAP is a central marker of AD: higher levels of GFAP in cortical gene and protein expression are associated with increased β-amyloid pathology, cerebral amyloid angiopathy, and faster cognitive decline, particularly in β-amyloid-positive individuals. In contrast, GFAP is not associated with tau pathology in the absence of amyloid nor with changes in the caudate nucleus [198]. Plasma GFAP can serve as a sensitive, non-invasive early marker of amyloid-β–induced astrogliosis. Plasma GFAP levels are elevated in β-amyloid–positive individuals and in those with mild cognitive impairment (MCI), regardless of cognitive status, and correlate with higher β-amyloid PET signal, even after controlling for tau [199,200]. Plasma GFAP appears to have better predictive power for amyloid positivity than CSF GFAP and other glial markers and is associated with both cognitive decline and tau accumulation over time [199]. Clinical trials targeting preclinical AD recruitment of β-amyloid-positive individuals with high GFAP levels could improve cost-effectiveness [201]. The transition from CSF to plasma analysis may facilitate molecular phenotyping of AD. Examining a biomarker panel (Aβ42/40, p-tau181, p-tau231, t-tau, NF-L, GFAP, UCHL-1, SNAP-25) in matched CSF and plasma samples across the AD continuum revealed strong correlations for Aβ42/40, p-tau181, p-tau231, NF-L, and GFAP between the two biofluids, whereas t-tau and UCHL-1 showed no correlation [202]. The best biomarkers for distinguishing controls from preclinical AD were p-tau231 and SNAP-25 in CSF and Aβ42/40, p-tau231, and GFAP in plasma. Moreover, GFAP, NF-L, and p-tau181 were significantly associated with disease progression in both biofluids, suggesting that a standardized plasma panel could support early diagnosis, staging, and monitoring of AD progression, even in preclinical stages [202].

### 5.4. Huntington’s Disease

HD is a single-gene neurodegenerative disorder exhibiting autosomal dominant inheritance that primarily affects the striatum and manifests with progressive motor, cognitive, and psychiatric symptoms appearing in adulthood. The underlying cause is an expansion of the CAG trinucleotide repeat in the gene encoding mutant huntingtin, which leads to the formation of intracellular aggregates that interfere with axonal trafficking and cytoskeletal dynamics [203]. At the same time, in neurons of HD model mice, mitochondria that are normally long and interconnected become short, isolated, swollen, with disorganized cristae. The overall mitochondrial network becomes fragmented, leading to diminished mitochondrial function, elevated ROS, and impaired mitophagy [204]. In these cases, although “breakdown” of IFs is not always highlighted (as in ALS), the recurring event is that cytoskeletal disorganization and the inability of neuronal and glial cells to manage oxidative stress, organelle transport, and intercellular communication—emerging factors in HD—and mitochondrial stress proceed in parallel during neuronal degeneration. Dysfunction of neurofilaments can lead to impaired axonal transport and reduced axonal caliber, promoting huntingtin accumulation within neurites and early axonal degeneration. Recent studies have shown that levels of NF-L in plasma and CSF correlate with disease state in HD, making NF-L one of the most promising biomarkers [205]. An increasing number of studies report a significant glial response in HD, with elevated density of GFAP-positive astrocytes in the cortex of affected individuals and in murine models [206,207]. In a study by Brown et al. [208], a heterogeneous spatial distribution of GFAP-positive astrocytes was observed in the zQ175 mouse model, with preferential accumulation in the dorsomedial striatum. These findings suggest that remodeling of the GFAP network may contribute to glial reorganization and influence the neuronal microenvironment in HD. Unlike Nfls and GFAP, the role of peripherin in HD remains largely unexplored. Although peripherin is known to play roles in peripheral axons and motor neurons, there are currently no robust data directly linking it to HD pathogenesis, representing an important gap in literature. Indeed, while the mechanistic links between IFs and mitochondria are well defined in ALS, evidence in PD and especially HD remains more correlative, relying primarily on biomarker studies such as NF-L and GFAP. This disparity reflects a gap in the field. In HD, an emerging area of interest concerns peripherin, rich in basic residues, which may modulate membrane surface proton capacity and promote non-bilayer lipid structures that could, in principle, mitigate bioenergetic deficits relevant to PD and HD pathology [209,210]. Clarifying whether such IF-dependent biophysical mechanisms operate in vivo represents relevant future directions.

**Table 1:** Main studies describing intermediate filament/cytoskeleton disorganization and mitochondrial defects in neurodegenerative diseases.

Disease	Cytoskeletal/IFs Alterations and Protein Aggregates Fromation	Mitochondrial Defects	Reference
Amyotrophic lateral sclerosis	Overexpression of peripherin; Slowed neurofilament transport; IF inclusions that precede axonal spheroids	Mitophagy defects; Lysosomal alterations; Accumulation of damaged organelles; Impaired mitochondrial turnover	[155,166]
Parkinson’s disease	Aggregation of α-synuclein; Alters cell morphology and likely associated cytoskeleton (microtubules, secondary IFs)	Reduced mitochondrial respiration; Oxidative stress; Membrane potential loss; Impaired mitochondrial quality	[183]
Alzheimer’s disease	Pathological tau spreads and interacts with microtubules and secondary IFs; Intracellular transport of organelles and proteins impaired by tau and Aβ aggregates	Mitochondrial transport defects in dendrites; Mitochondrial fragmentation; Dynamin-related protein 1 activation; Increased reactive oxygen species	[195]
Huntington’s disease	Mutant huntingtin aggregates; Accumulation around inclusions; Interference with cytoskeletal trafficking; Cytoskeletal disorganization implicated in transport block	Reduced mitochondrial mobility; Altered morphology (cristae, size); Fragmented mitochondrial network	[203]

## 6. Conclusions

Mitochondrial failure and dysregulated IF dynamics may represent a convergent pathogenic axis in neurodegeneration. Progressive decline in MQC compromises neuronal bioenergetics and resilience, while remodeling of IF networks, through altered expression, phosphorylation, or aggregation, may further aggravate oxidative stress and organelle mislocalization. These reciprocal perturbations suggest that cytoskeletal integrity and mitochondrial health are tightly coupled. However, several key questions remain open and warrant systematic exploration. For instance, although proteins such as plectin and MFN2 have been implicated in bridging IFs to mitochondria, the precise molecular determinants of direct IF–mitochondria binding are not fully defined. High-resolution interactomics and structural studies will be essential to delineate the specific interfaces and their regulation under physiological and stress conditions. Moreover, whether it is possible to therapeutically modulate IF phosphorylation, assembly, and/or turnover to improve mitochondrial positioning and function, without compromising the cytoskeletal integrity required for neuronal stability, is currently unknown. The emerging use of IF-derived proteins such as peripherin and GFAP as circulating biomarkers holds diagnostic potential, but methodological standardization and clinical validation are needed. Establishing quantitative thresholds, temporal profiles, and disease specificity will be critical to integrate these proteins into biomarker panels for ALS, PD, AD, and related disorders. Future research should also investigate how IFs coordinate mitochondria–ER contacts, lysosomal function, and mitophagy, potentially linking cytoskeletal remodeling to broader organelle network homeostasis. Altogether, integrating molecular, imaging, and clinical approaches will clarify how IF–mitochondria interactions can be leveraged for early diagnosis and intervention in neurodegenerative diseases. Therapeutic strategies that restore mitochondrial integrity, modulate IF dynamics, or disrupt maladaptive IF–mitochondria coupling hold strong potential and warrant investigation.

## Data Availability

No new data were created or analyzed in this study. Data sharing is not applicable to this article.
